# Diode laser photoacoustic spectroscopy of CO_2_, H_2_S and O_2_ in a differential Helmholtz resonator for trace gas analysis in the biosciences and petrochemistry

**DOI:** 10.1007/s00216-019-01877-0

**Published:** 2019-05-20

**Authors:** Saeed Alahmari, Xiu-Wen Kang, Michael Hippler

**Affiliations:** Department of Chemistry, University of Sheffield, Sheffield, S3 7HF UK

**Keywords:** Gas sensors, IR/Raman spectroscopy, Optical sensors, Bioanalytical, Petrochemistry

## Abstract

Photoacoustic spectroscopy in a differential Helmholtz resonator has been employed with near-IR and red diode lasers for the detection of CO_2_, H_2_S and O_2_ in 1 bar of air/N_2_ and natural gas, in static and flow cell measurements. With the red distributed feedback (DFB) diode laser, O_2_ can be detected at 764.3 nm with a noise equivalent detection limit of 0.60 mbar (600 ppmv) in 1 bar of air (35-mW laser, 1-s integration), corresponding to a normalised absorption coefficient *α* = 2.2 × 10^−8^ cm^−1^ W s^1/2^. Within the tuning range of the near-IR DFB diode laser (6357–6378 cm^−1^), CO_2_ and H_2_S absorption features can be accessed, with a noise equivalent detection limit of 0.160 mbar (160 ppmv) CO_2_ in 1 bar N_2_ (30-mW laser, 1-s integration), corresponding to a normalised absorption coefficient *α* = 8.3 × 10^−9^ cm^−1^ W s^1/2^. Due to stronger absorptions, the noise equivalent detection limit of H_2_S in 1 bar N_2_ is 0.022 mbar (22 ppmv) at 1-s integration time. Similar detection limits apply to trace impurities in 1 bar natural gas. Detection limits scale linearly with laser power and with the square root of integration time. At 16-s total measurement time to obtain a spectrum, a noise equivalent detection limit of 40 ppmv CO_2_ is obtained after a spectral line fitting procedure, for example. Possible interferences due to weak water and methane absorptions have been discussed and shown to be either negligible or easy to correct. The setup has been used for simultaneous in situ monitoring of O_2_, CO_2_ and H_2_S in the cysteine metabolism of microbes (*E*. *coli*), and for the analysis of CO_2_ and H_2_S impurities in natural gas. Due to the inherent signal amplification and noise cancellation, photoacoustic spectroscopy in a differential Helmholtz resonator has a great potential for trace gas analysis, with possible applications including safety monitoring of toxic gases and applications in the biosciences and for natural gas analysis in petrochemistry.

**Figure:**
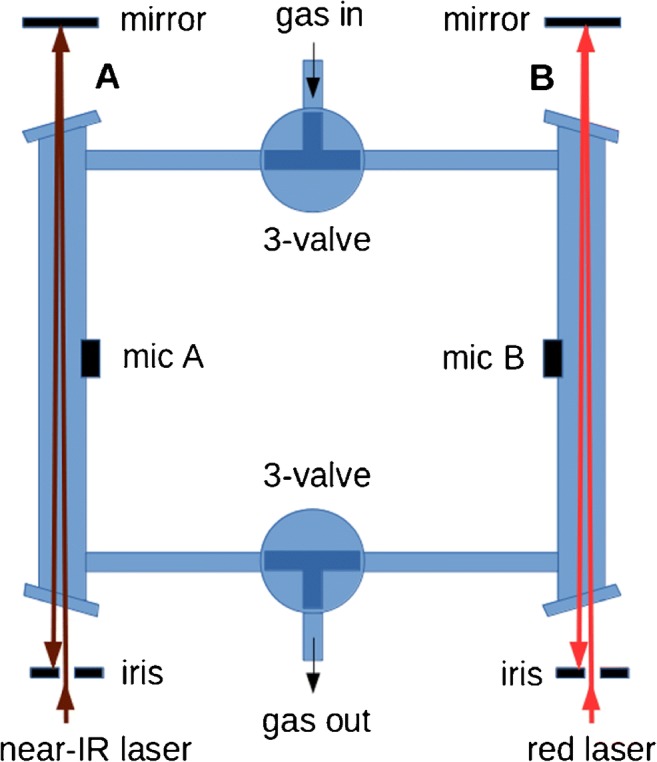
Graphical abstract

Graphical abstract

## Introduction

Trace gas detection is essential in many areas of fundamental and applied research, including environmental monitoring, industrial process control and biological applications. In this context, the detection of CO_2_, O_2_ and H_2_S is particularly relevant. Carbon dioxide (CO_2_) is the main anthropogenic greenhouse gas with a current ambient level of 410 ppmv in air. It is also a main product of the metabolism of organisms. The ability of an analytical technique to distinguish isotopes will allow isotope labelling experiments to determine sources and sinks of CO_2_. In the urea breath test for *Helicobacter pylori* (*H*. *pylori*), for example, patients swallow ^13^C-labelled urea. ^13^CO_2_ detected in the exhaled breath indicates *H*. *pylori* infection in the stomach, since only this bacterium can digest urea efficiently to release ammonia and carbon dioxide. CO_2_ detection is particularly relevant to study the metabolism and activity of bacteria. Monitoring of molecular oxygen (O_2_) complements such metabolism studies. O_2_ detection is also very relevant in biotechnology, for example to ensure that there is no oxygen in a bioreactor in anaerobic fermentation processes. Hydrogen sulfide (H_2_S) is a very toxic gas which is comparable in toxicity with carbon monoxide [1–3]. It has an immediately dangerous to life or health (IDLH) limit of 100 ppmv in air. It is therefore important to be able to monitor H_2_S with great sensitivity and selectivity as a toxic industrial and environmental compound. This is particularly relevant in petrochemistry since H_2_S is a common minor component in natural gas, but due to its high toxicity, it has been removed at source before the gas can be fed to gas supply lines [2–4]. H_2_S is also relevant in biochemistry and microbiology, with an important role as a signalling molecule as well as a cytoprotectant [5–7]. It has been recently reported that H_2_S helps defend a number of bacteria against antibiotics, including *Escherichia coli* (*E*. *coli*) [7]. Understanding the mechanisms of its production by microbes may therefore be very relevant to develop new strategies to protect and enhance the potency of existing antibiotics. Although bacteria are known to produce the gas during the metabolism of sulfur compounds, the biochemistry associated with these processes is not fully understood. In many organisms, H_2_S is generated during degradation of the sulfur-containing amino acid l-cysteine. Cysteine desulphhydrases are enzymes which convert cysteine to pyruvate, a source of energy and a key intermediate in the production of ATP. This process releases one equivalent of ammonia and hydrogen sulfide [7, 8]. In microbiology, a common test for H_2_S is the lead acetate or the methylene blue assay; although very sensitive, these assays can be difficult to apply for quantitative measurements and may suffer from interferences. Common analytical techniques include gas chromatography (GC) or mass spectrometry (MS); whilst sensitive and selective, they require expensive equipment and have limitations, including difficulties detecting certain components, long analysis times for GC, and the need for sample preparation which prevents real-time, in situ monitoring. Solid-state electrochemical and chemisorbing sensors, where a change in physical properties on adsorption of analyte gas molecules is detected, or acoustic sensors which measure changes in acoustic properties of gas mixtures are also widely used [9, 10]; although detection limits in the ppm range can be achieved, these sensors often suffer from several disadvantages. Chemisorbing sensors, for example, are often affected by ageing and poisoning of the sensor surfaces, from long response and settlement times, and from interferences due to limited selectivity. Acoustic sensors have a response that depends on temperature, and the method lacks selectivity.

In trace gas analysis, spectroscopic detection has distinct advantages due to its quantitative nature and its sensitivity and selectivity due to the very characteristic spectroscopic signature of different molecules. Detection by optical absorption is possible for CO_2_ and H_2_S using strong fundamental vibrational bands in the mid-IR, or much weaker overtones and combination bands in the near-IR. Although weaker, near-IR absorption offers the advantages of much more convenient optics and light sources in the near-IR. Molecular oxygen (O_2_) does not have an IR-active vibration; although Raman detection of its fundamental is possible, Raman scattering is a rather weak process and special Raman enhancement techniques are required to detect gases at the ppmv level, for example by stimulated Raman photoacoustic spectroscopy (PARS) [11] or the recently introduced technique of cavity-enhanced Raman spectroscopy (CERS) [4, 12–14]. O_2_ can be detected by a formally forbidden electronic absorption band in the red, the *b*^1^Σ_g_^+^ (*ν* = 0) ← *X*^3^Σ_g_^−^ (*ν* = 0) band (the “*A* band”) near 760 nm; although weak, the absorption cross sections are comparable to near-IR overtone and combination band absorptions of CO_2_ and H_2_S. For efficient detection beyond the limitations of conventional, Beer-Lambert-type absorption, absorption pathlengths should be as long as possible, as in the extreme case of cavity ringdown spectroscopy which achieves kilometre effective pathlengths [15–19], or detection should be coupled with a background-free scheme as in photoacoustic spectroscopy [20–27], or a combination of both as in the recently introduced technique of cavity-enhanced resonant photoacoustic spectroscopy (CERPAS) [28, 29]. In photoacoustic spectroscopy, internal excitation of molecules by optical absorption is converted to heat release and pressure increase by collisions; if the excitations are modulated periodically, acoustic waves are induced which can be picked up by microphones. This allows indirect, but very sensitive detection of optical absorption. By detecting in an acoustic resonator and modulating optical excitation at an acoustic resonance frequency, building up of acoustic standing waves further increases sensitivity in resonant photoacoustic spectroscopy [20, 21]. Highest sensitivities are achieved with CERPAS [28, 29] or utilising the excitation of the acoustic modes of a quartz tuning fork (quartz-enhanced or cantilever-enhanced photoacoustic spectroscopy) [26, 27]. A much simpler approach is employing a special acoustic resonator which effectively amplifies signal and reduces noise in a differential Helmholtz resonator (DHR) [22–25].

In this contribution, we report photoacoustic trace gas monitoring in a differential Helmholtz resonator. Photoacoustic detection is one of the most sensitive optical absorption techniques, but it suffers from ambient acoustic noise and flow noise introduced by sampling gases; this ultimately limits detection limits. A DHR has the advantage that the symmetrical resonator composed of two identical chambers produces photoacoustic absorption signals which are out-of-phase in the two chambers, whereas noise including flow noise is in phase in the two chambers. Differential detection therefore doubles the signal and cancels noise to a great extent. DHR photoacoustic spectroscopy has been reported before for methane detection [23]; however, the power of the specific configuration and the underlying principles are not fully appreciated in our opinion. Herein, we demonstrate the capabilities of the technique with three different new and relevant target analytes, trace gas detection of CO_2_ and H_2_S in a DHR with distributed feedback (DFB) diode lasers near 1.6 μm, and O_2_ detection near 760 nm. Improvements on the DHR technique are reported, including a multipass arrangement and using two independent lasers. We also introduce novel applications of DHR detection in the biosciences and in petrochemistry, including time-dependent monitoring of the bacterial growth and aerobic metabolism of microbes, and detection of H_2_S and CO_2_ impurities in natural gas.

## Experimental

**Fig. 1:**
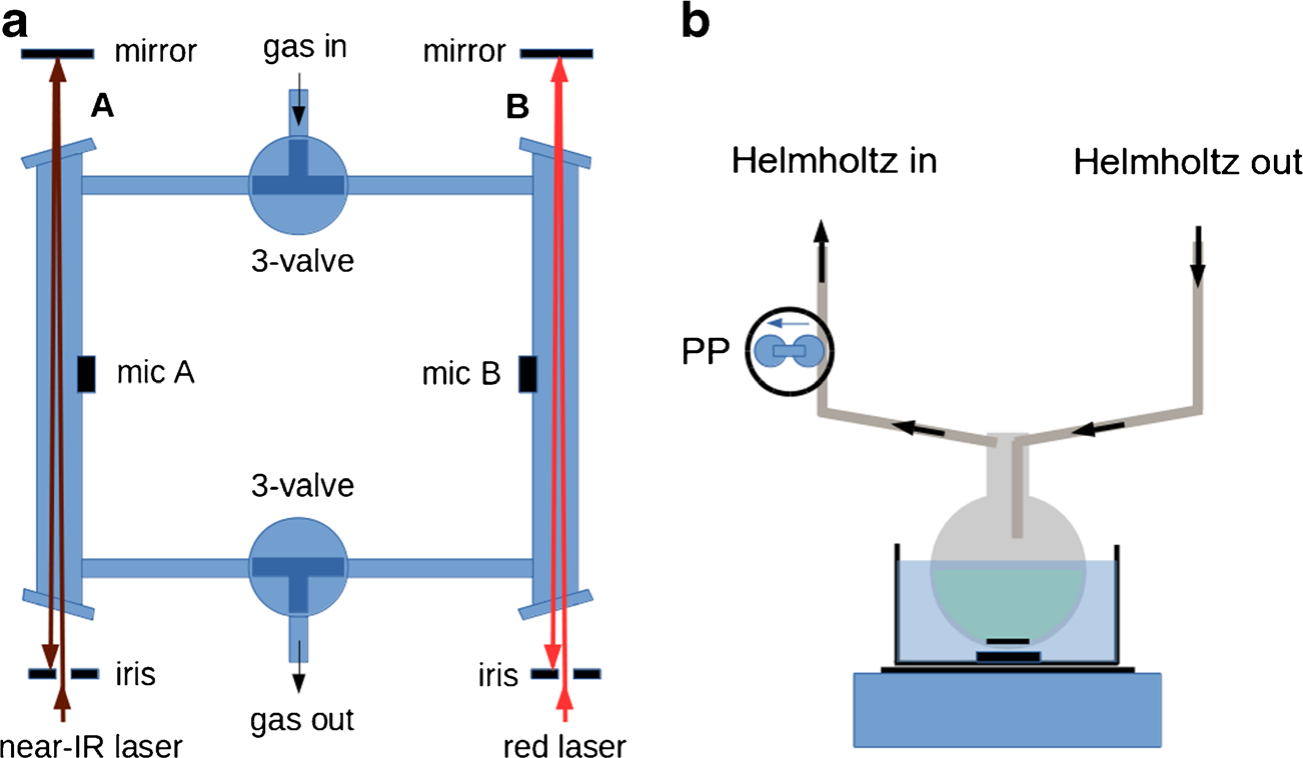
Scheme of the experimental setup. **a** Differential Helmholtz resonator with the two laser beams (see main text for more details). **b** In biological experiments, the head space of a round-bottom flask in a thermostated water bath is circulated to the Helmholtz resonator via a peristaltic pump (PP)

The differential Helmholtz resonator in the present experiment consists of two parts which are made as symmetrical as possible (see Fig. 1a). It follows a design recently published in ref. [23]. It has two identical cylindrical compartments (A and B in Fig. 1a) made of glass, 10 cm long and 1 cm inner diameter (i.d.). The compartments are connected by two capillary glass tubes, 10 cm long and 0.2 cm i.d. Each compartment has in the middle one electret microphone (Knowles EK-23024) attached to it from the outside, but open to the inside. The compartments have glass windows at their end to allow laser light passing through; the windows are slightly tilted to avoid back reflections into the laser. The thin connecting tubes have each a three-way valve in the middle which allows either to separate the Helmholtz resonator for static measurements, or to have a symmetrical gas flow through both compartments in a flow cell configuration. The entire cell is wrapped in heating wire and thermal insulation to keep it at around 45 °C, to avoid water condensation in biological experiments. Diode laser light passes through one of the compartments, e.g. A in Fig. 1a, and is reflected back once at a slight angle to double the interaction path length. An iris acts as a backstop for the reflected beam. Whereas for an organ pipe resonator the laser beam would have to be focussed into the middle of the resonator to enhance the longitudinal acoustic mode, focussing is not required for this DHR resonator since the acoustic mode extends over the entire compartment (see below). This has the advantage that no refocussing is required, and a simple mirror is enough to double the interaction beam path; we have confirmed that this simple arrangement indeed increases photoacoustic signal by about a factor of 2.

After the laser light is absorbed by molecules inside compartment A, collisional deactivation leads to a temperature jump and pressure expansion (photoacoustic effect). This pressure buildup will then travel to compartment B via the connecting capillaries. If the laser light is pulsed, the pressure buildup will travel from A to B, leaving a pressure depression behind in A, and then back again to A when the laser is off, leaving a pressure depression behind in B. If the laser is modulated periodically, periodic pressure waves (sound) are thus created in A and B, which have the same frequency and amplitude, but opposing phases. If the laser modulation matches a resonance frequency of the cell, a standing wave develops with maximum amplitude (resonant photoacoustics). Note that the acoustic resonance is a pressure oscillation between A and B, not a longitudinal acoustic resonance as in the more commonly used organ pipe photoacoustic resonators. The resonance frequency is given by the cell dimensions, and it depends also linearly on the speed of sound *c*, which for an ideal gas is given by Eq. 1,

1 $$ c=\sqrt{\frac{\gamma RT}{M}}, $$

where *T* is the temperature, *R* the gas constant, and γ and *M* the heat capacity ratio and the molar mass of the medium inside the resonator, respectively. Using a CO_2_ absorption line in the near-IR, we obtained a resonance curve (amplitude vs. acoustic frequency). The data are well described by a Lorentzian distribution with resonance frequency *f* = 220 Hz for 1 bar CO_2_. In an atmosphere of air or N_2_, *f* increases to 261 Hz, and in natural gas (essentially CH_4_) up to 320 Hz, consistent with the changes in molar masses. The quality factor *Q* is defined as the ratio between the resonance frequency *f* and the frequency bandwidth at 1/√2 of the maximum of the resonant profile. For our DHR, *Q* ≈ 7 is determined. *Q* is roughly equivalent to the signal enhancement by the acoustic resonance. Due to the changes in resonance frequency with temperature and medium, a calibration is strictly valid only for a given temperature and gas composition. However, due to the relatively low *Q* factor, slight misalignment of the resonance frequency does not have a large effect on signals. The change in composition from trace gas levels to higher levels will cause a slight curvature of the calibration instead of linearity; this is considered by using a calibration curve instead of a calibration line (see also discussion below). Note that the selectivity of photoacoustic detection is from the spectroscopic signature of gases within a composition, and not from the change in acoustic properties.

**Fig. 2:**
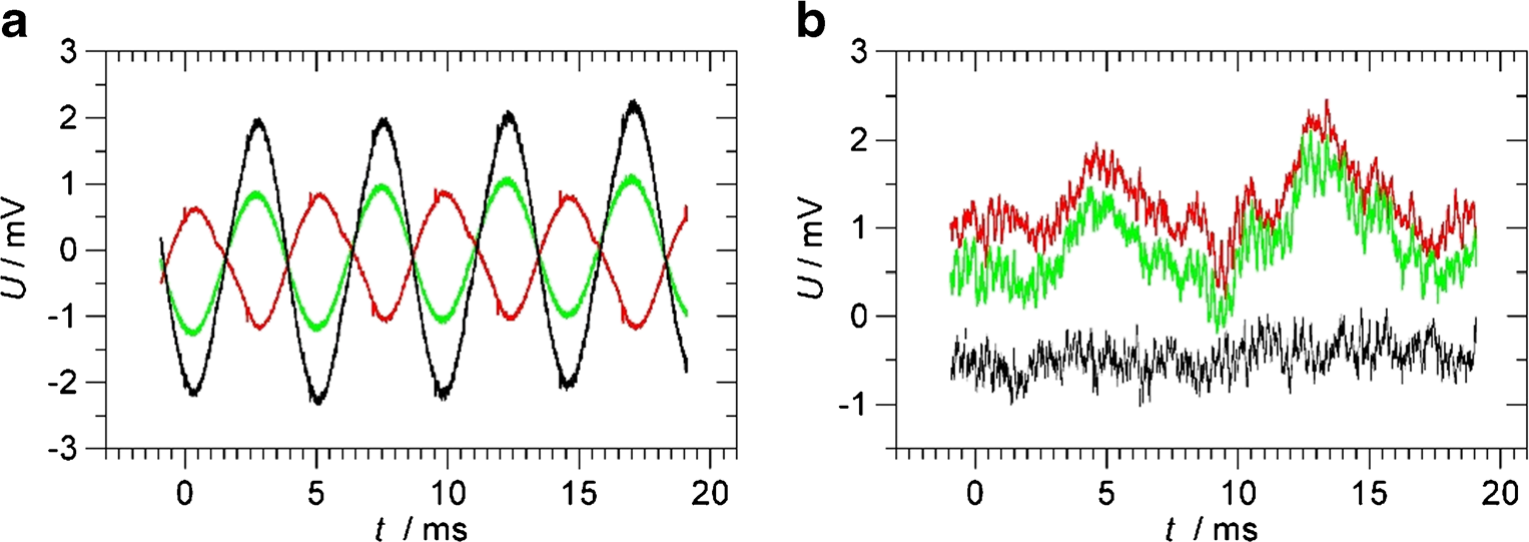
Oscilloscope traces of the signal from microphone A (green) and microphone B (red) and the differential signal A–B (black). **a** Signal enhancement (CO_2_ absorption near 1.57 μm). **b** Noise cancellation (flow noise in a flow cell configuration) (see main text for more detail)

Characteristic features of a DHR are its low resonance frequency and effective noise cancellation and signal enhancement by differential amplification of microphone signals A–B. Genuine absorption signals inside the cell are out-of-phase between microphones A and B; differential amplification A–B therefore doubles the signal (see Fig. 2a). External noise and flow noise, however, will affect the two symmetrical compartments in nearly the same way, creating a noise signal which is in phase in A and B. In this case, differential amplification A–B leads to effective cancellation of noise (see Fig. 2b). This noise cancellation and signal enhancement make DHR an attractive choice for trace gas detection applications. The differential signals (A–B) are processed in lock-in amplifiers which further greatly reduce noise.

In our setup, two DFB diode lasers are used, i.e. a near-IR laser to detect CO_2_ and H_2_S near 1.57 μm and a red laser near 764 nm to detect O_2_. To simplify the setup, the near-IR laser is directed through one compartment (A), whilst the red laser is directed through the other (B) (see Fig. 1a). In a typical experiment, CO_2_ and H_2_S are first measured by scanning the near-IR laser; next, O_2_ is measured by scanning the red laser. Note that during a scan with one laser, only this laser is on whilst the other is switched off. Both lasers are modulated by their injection current at the acoustic resonance frequency with a square wave giving a 50% duty cycle. The near-IR single-mode DFB diode (Mitsubishi FU-650SDF, 4 mW) is amplified in a booster optical amplifier (Thorlabs S9FC1004P) to 30 mW peak power. The laser is temperature tuneable between 20 and 60 °C to provide a mode-hop-free tuning range from 6378 to 6357cm^−1^ or from 1.568 to 1.573 μm. The red laser is a temperature tuneable 35-mW DFB diode laser (Eagleyard EYP-DFB-0764); adjusting the temperature from 18 to 40 °C gives a 764.0–765.3-nm mode-hop-free tuning range.

For bacterial measurements, 50 ml of sterile 2YT medium (a rich medium containing yeast extract and tryptone) was inoculated with a single colony of *E*. *coli* (wild type, strain K-12 MG1655) and incubated for 5 h at 37 °C. It was added to 50 ml of sterile 2YT to give a 100-ml suspension in a 500-ml round-bottom flask with constant stirring at 37 °C in a thermostated water bath. The flask was connected to the DHR resonator with short gas transfer tubes in a closed system, giving a total gas volume of 660 ml. To enhance gas flow, a peristaltic pump (3 l/h) was used to cycle the flask head space through the DHR resonator (see Fig. 1b). In a test to characterise the experimental time resolution, CO_2_ or H_2_S gas was injected into the flask and the appearance time of the gas was measured in the Helmholtz resonator; equilibrium is reached within about 5 min. To observe O_2_, CO_2_ and H_2_S in the aerobic respiration of *E*. *coli*, a cysteine solution (l-cysteine hydrochloride anhydrous, Sigma) dissolved in 7 ml of water is injected via a septum (Suba-Seal) as a source of sulfur to induce the production of H_2_S. At the beginning of an experiment, the suspension has typically OD_600_ ≈ 0.6 (optical density at 600 nm in a 1-cm cuvette) and a pH of 6.9; at the end, after exhaustion of the oxygen available, the suspension has typically OD_600_ ≈ 2.2 and a pH of 7.2.

Calibrated gas mixtures containing CO_2_ (sublimed from dry ice) or H_2_S (Sigma-Aldrich, 99.5+%) were prepared on a glass vacuum line equipped with capacitance pressure gauges (Baratron). The gases were purified by repeated freeze-pump-thaw cycles and buffered to 1 bar total pressure by N_2_, natural gas or air. Natural gas was sampled from a gas tap within the PC teaching laboratory of the University (gas supplied via National Grid, UK); natural gas is essentially CH_4_ with some additional minor components (see ref. [4] for typical compositions).

## Results and discussion

### Photoacoustic detection of molecular oxygen near 764 nm

**Fig. 3:**
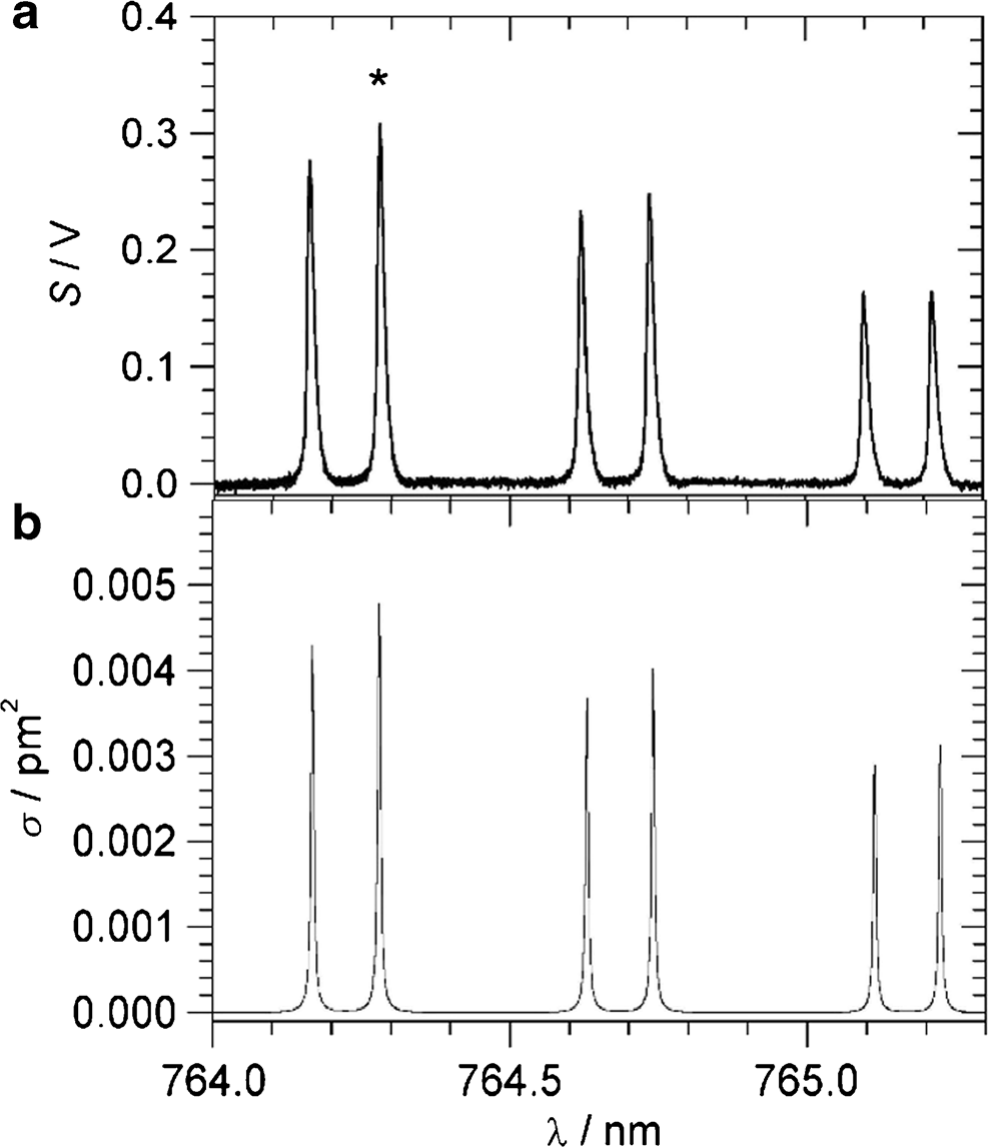
**a** Photoacoustic spectrum of 1 bar air in the Helmholtz resonator, obtained by temperature tuning the laser from 24 to 38 °C. The peak marked by the asterisk is used for the detection of molecular oxygen. **b** HITRAN absorption cross sections (1 bar air pressure broadening) of O_2_ in the *A* band [30]

Molecular oxygen (O_2_) does not have an IR-active vibration, but it can be detected by an electronic absorption band in the red, the *b*^1^Σ_g_^+^ (*ν* = 0) ← *X*^3^Σ_g_^−^ (*ν* = 0) band of molecular oxygen (the “*A* band”) near 760 nm. It is a very weak, formally forbidden transition with absorption cross sections of the same order as the near-IR combination bands of CO_2_ and H_2_S near 1.57 μm discussed later. Some of the strongest rotational lines of the *A* band are within the range of our DFB diode laser. Figure 3 shows the photoacoustic spectrum of 1 bar air obtained in the Helmholtz resonator, by temperature tuning the laser from 24 to 38 °C. A good agreement with literature data (HITRAN) is obtained [30]. The strongest line is used for the analysis of O_2_; it is marked by an asterisk in Fig. 3. The transition is the *P*(11) line at 764.280 nm (vacuum) which has a peak absorption cross section of *σ*_peak_ = 0.00478 pm^2^ (1 bar pressure broadened) [30].

At an abundance of 210 mbar in 1 bar air, the peak photoacoustic signal of this line is 310 mV. The standard deviation (*σ*) of the baseline, 0.88 mV at 1-s integration time, can serve as an estimate of the noise level. A noise equivalent detection limit of 0.60 mbar (600 ppmv) or 0.06% of O_2_ in 1 bar of air at 35-mW peak power and 1-s integration time is thus obtained. Note that this is the *noise equivalent* detection limit (1 *σ* limit); practical detection limits are often quoted as 3 *σ* in the literature. Since photoacoustic signal scales linearly with laser power and statistical noise decreases with the square root of integration time (see also below), improved detection limits are achieved with higher laser power and longer integration times. Normalising to the absorption cross section, the laser power and integration time, a noise equivalent *normalised* absorption coefficient *α* = 2.2 × 10^−8^ cm^−1^ W s^1/2^ is calculated. Considering the simplicity of the DHR setup, this compares favourably with the detection limits of more complex photoacoustic schemes, such as quartz–cantilever-enhanced photoacoustic spectroscopy where *α* = 4.8 × 10^−9^ cm^−1^ W s^1/2^ was reported for photoacoustic detection of oxygen by a 30-mW DFB diode near 760 nm [26]. In an application where the head space above an aqueous solution is measured (as in the bacterial suspensions at 37 °C, see “Monitoring O_2_, CO_2_ and H_2_S during the metabolism of l-cysteine by E. coli”), 600 ppmv of O_2_ in the gas phase corresponds to an oxygen concentration of 12 ppb per mole in the solution, using Henry’s law and Henry’s constant at 37 °C [31].

### Photoacoustic detection of H_2_S and CO_2_ in air/N_2_ near 1.57 μm

**Fig. 4:**
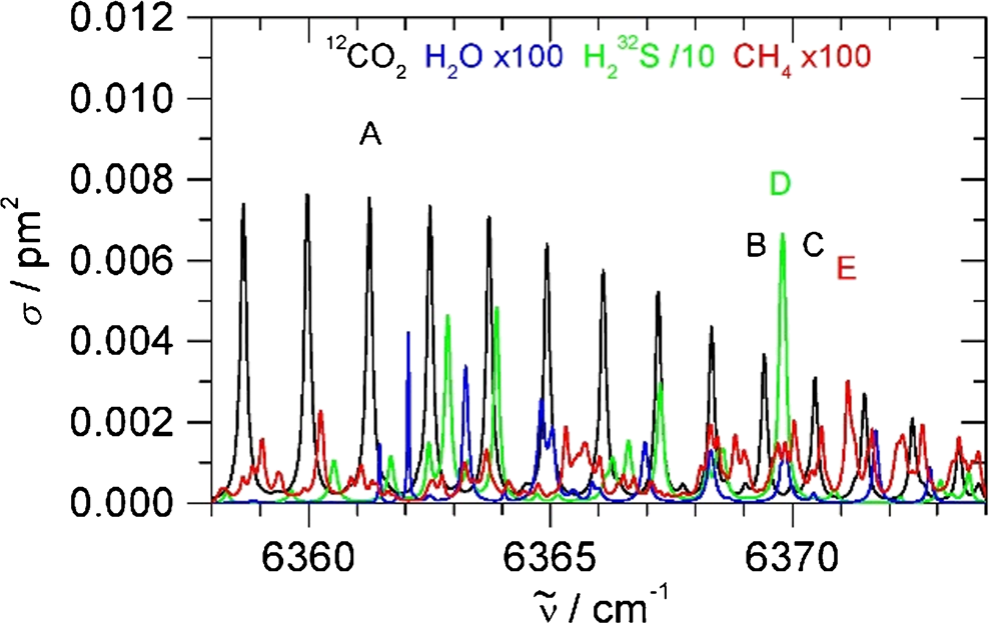
HITRAN absorption cross sections (1 bar air pressure broadening) of CO_2_ (black), H_2_S (green, scaled × 0.1), water vapour (blue, scaled × 100) and methane CH_4_ (red, scaled × 100) [30]. Lines A to C are CO_2_ peaks, D is a H_2_S peak and E is a CH_4_ peak discussed in the main text

**Fig. 5:**
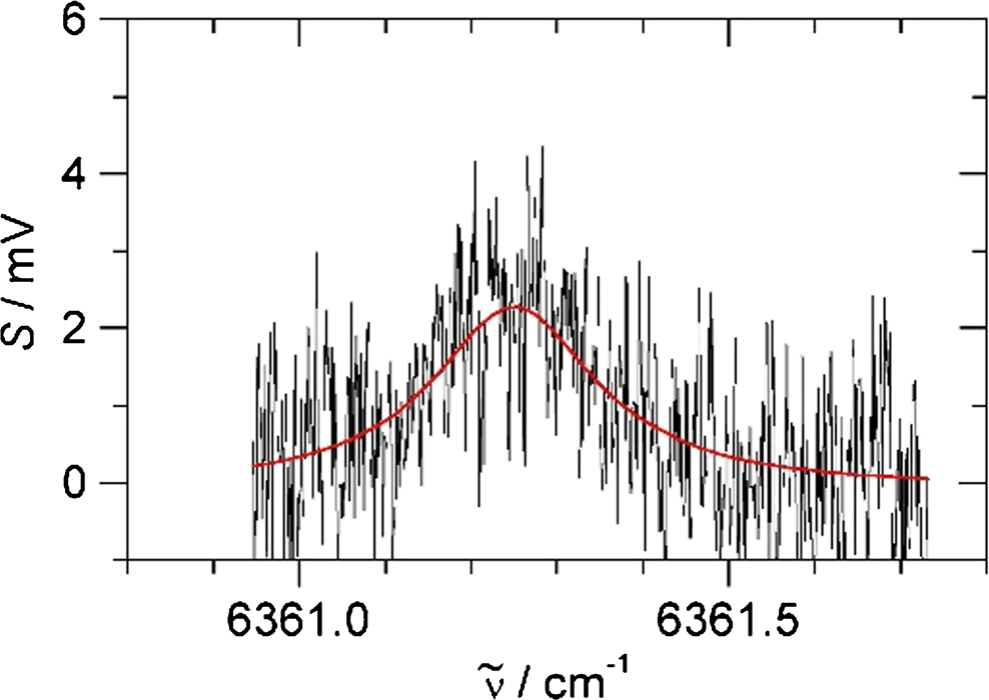
Photoacoustic spectrum of CO_2_ at natural abundance in 1 bar of ambient air. Red: Lorentzian line fit

In this section, we aim for the detection of CO_2_ and H_2_S in air or N_2_. Within the tuning range of the near-IR DFB diode laser (6357–6378 cm^−1^), CO_2_ and H_2_S absorption features can be accessed which can be used for trace gas detection. Figure 4 provides an overview from the HITRAN database including the effect of 1 bar air broadening [30]. CO_2_ shows part of the *R*-branch of the 3ν_1_ overtone, with a characteristic nuclear spin statistic which only allows transitions from even *J*. The strongest line (labelled A in Fig. 4) is the *R*(18) line at 6361.250 cm^−1^ with peak absorption cross section *σ*_peak_ = 0.00757 pm^2^ (1 bar air pressure broadened) and Lorentzian line shape [30]. This line was used for CO_2_ detection. H_2_S has unresolved or partially resolved features in the region. The strongest is a feature at 6369.8 cm^−1^ of the ν_1_ν_2_ν_3_ combination band (labelled **D** in Fig. 4). This feature was employed for H_2_S detection. Its peak absorption cross section is *σ*_peak_ = 0.0665 pm^2^ (1 bar air pressure broadened), about nine times stronger than the CO_2_ line [30]. Figure 4 also displays water absorptions to assess whether water will interfere with CO_2_ or H_2_S detection. Water quite often interferes very severely in “real-life” applications, in particular in the region of mid-IR fundamental vibrations. Fortunately, the absorption features of water are very weak in this near-IR region, with absorption cross sections below 4 × 10^−5^ pm^2^ [30]. The peaks chosen are well isolated with negligible interference in air (natural gas is discussed separately below). To demonstrate the sensitivity of the DHR scheme, ambient air from the outside was measured (see Fig. 5). At 1-s integration time of each data point, the *R*(18) line due to CO_2_ at natural abundance (410 ppmv) can clearly be distinguished.

**Fig. 6:**
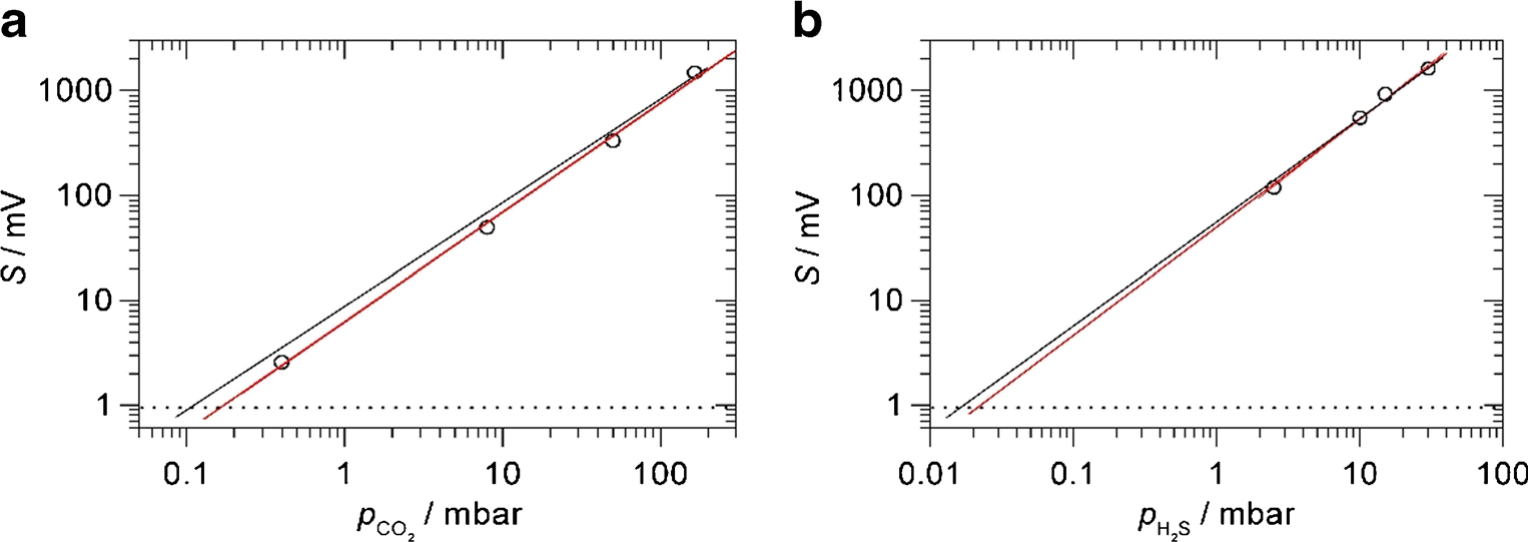
Calibration plots. Black: linear fit through origin, *y* = *a x*. Red: non-linear fit, *y* = *a x*^*b*^. Horizontal, dotted line: noise floor at 1-s integration time. **a** Observing the 6361.250-cm^−1^*R*(18) line of CO_2_ in 1 bar N_2_. **b** Observing the 6369.8-cm^−1^ feature of H_2_S in 1 bar N_2_ (see main text for more detail)

In order to characterise the system, calibrated gas mixtures of CO_2_ in 1 bar N_2_ were prepared and the *R*(18) line was measured by DHR photoacoustics. The amplitude of the peak vs. CO_2_ partial pressure is shown in the calibration plot of Fig. 6a. Good linearity is obtained (black fit line; *y* = *a x*), although a slightly better fit is obtained by a non-linear fit to *y* = *a x*^*b*^ (red fit line) with *b* = 1.04. However, the deviation from linearity is not very pronounced. The deviation from linearity is probably caused by the change in the physical properties (e.g. speed of sound) in the mixture, from 200 mbar CO_2_ in 1 bar N_2_, to essentially just traces of CO_2_ in 1 bar N_2_. At 1-s integration time, the standard deviation of the baseline is about 0.98 mV with the laser still on; this level is indicated by the horizontal, dotted line. It represents the noise floor at 1-s integration time. The non-linear red fit line hits the noise level at 0.160 mbar or 160 ppmv CO_2_ in 1 bar N_2_, giving the noise equivalent detection limit at 1-s integration time with the 30-mW laser. Normalising to absorption cross section, laser power and integration time, a noise equivalent normalised absorption coefficient *α* = 8.3 × 10^−9^ cm^−1^ W s^1/2^ is calculated. Again, considering the simplicity of the DHR setup, this compares favourably with more involved photoacoustic schemes, such as the quartz–cantilever-enhanced photoacoustic detection reported with *α* = 2.2 × 10^−9^ cm^−1^ W s^1/2^ at 1572 nm [27].

Better detection limits are obtained by longer integration times or by fitting spectral line shapes to several data points in a spectrum. This is demonstrated in Fig. 5, for example, where 410 ppmv of CO_2_ in ambient air is clearly detectable despite a noise equivalent limit of 160 ppmv. A spectral line fit of a spectral scan is preferable to having a longer integration time on a single point, because the fit also gives the baseline/background value above which the signal is measured. Following a procedure described in ref. [11], if measurements are made in a reproducible way, there is an a priori knowledge about the expected peak position and the expected line width of the peak. In a Lorentzian line fit of spectral data, the line position and full width at half maximum (FWHM) can be kept fixed based on values obtained in measurements at higher concentration. The only free parameters of the fit are the offset and the peak height. The fitting is insensitive to slow variation of the baseline, removing the need for data manipulation. This restricted spectral fitting procedure greatly increases sensitivity and selectivity and removes the arbitrariness of baseline manipulations [11]. Using 16 data points spread around the central *R*(18) line within a range of 3 Lorentzian FWHM gives a good representation of the spectral peak to be analysed. Sixteen data points at 1-s integration time require a total measurement time of 16 s. The restricted fit of the gas mixtures used for the calibration line gives peak heights as in Fig. 6a). Repeated restricted fits of a gas mixture without CO_2_ (essentially a flat baseline with noise) give small positive and negative peak heights with a standard deviation (noise level) of 0.24 mV. The approximately fourfold improvement on the 0.98-mV noise level at 1-s integration agrees with the expectation that statistical noise decreases with the square root of integration time. The red, non-linear calibration of Fig. 6a) hits the 0.24-mV noise floor at 0.040 mbar CO_2_ in 1 bar N_2_, giving a 40 ppmv noise equivalent detection limit at 16-s measurement time.

In a similar calibration for H_2_S detection observing the 6369.8-cm^−1^ feature in 1 bar N_2_, the calibration in Fig. 6b) is obtained. The red fit curve shows again a slight non-linearity with *b* = 1.06. With the same noise limit of 0.98 mV at 1-s integration time, a noise equivalent detection limit of 0.022 mbar or 22 ppmv in 1 bar N_2_ is obtained with the 30-mW laser. The much lower detection limit compared to CO_2_ is due to the stronger absorption cross section of H_2_S. The detection limit is below the immediately dangerous to life or health (IDLH) limit of 100 ppmv; the DHR sensor may therefore be useful as a safety monitor for toxic levels of H_2_S, in particular if additional integration time or spectral line fitting procedures improve the detection limit (see above). At the same position of H_2_S peak **D**, there is also a very weak water peak with *σ*_peak_ = 1.5 × 10^−5^ pm^2^ [30], about 5000 times weaker than H_2_S. Assuming a similar sensitivity of photoacoustic signals, the noise limit will be exceeded only at water vapour pressures above 100 mbar, so this will not be a serious interference at ambient temperatures and water vapour pressures. In an application where the head space above an aqueous solution is measured (see next section), 160 ppmv of CO_2_ in the gas phase corresponds to a CO_2_ concentration of 76 ppb per mole in the solution, and 22 ppmv of H_2_S gas corresponds to 27 ppb in solution, using Henry’s law and Henry’s constants at 37 °C [31].

In previous work using mid-IR quantum cascade lasers (QCL), sub-ppm detection limits have been reported for IR-active molecules including H_2_S, in particular when combined with special techniques like frequency modulation (FM) absorption spectroscopy [32, 33]. In a possible extension, mid-IR QCLs could also be used in the DHR scheme to detect IR-active molecules via the stronger fundamental vibrational bands; based on our normalised absorption coefficients, sub-ppm detection limits are then comfortably predicted. The distinct advantages of our approach using near-IR laser and a photoacoustic scheme, however, are (i) convenience and lower costs; near-IR laser sources and detectors are in general much less than one tenth of the costs of a comparable QCL system; (ii) water interferences are much more of a concern in the mid-IR; and (iii) unlike photoacoustic spectroscopy, FM spectroscopy requires reduced pressure to work efficiently to minimise pressure broadening; this is a big disadvantage in the analysis of natural gas, for example, where gas lines are usually at atmospheric or higher pressures.

### Monitoring O_2_, CO_2_ and H_2_S during the metabolism of l-cysteine by *E*. *coli*

In a demonstration of an application in the biosciences, we monitored O_2_, CO_2_ and H_2_S during the metabolism of l-cysteine by *E*. *coli*. During aerobic metabolism, microbes convert O_2_ and sugars or other suitable organic substrates to CO_2_, with typically one unit of CO_2_ produced per unit O_2_ consumed. In addition, if l-cysteine is present, they may produce H_2_S, with approximately one unit of H_2_S produced per unit of cysteine consumed. In our closed system, O_2_, CO_2_ and H_2_S are mostly in the gas phase, with some minor amount dissolved in the suspension. The total amount in the gas phase can be calculated by the ideal gas law. At equilibrium, the molarity of a dissolved gas can be calculated from its partial pressure using Henry’s law. One millibar of an ideal gas corresponds to 2.6 × 10^−5^ mol in the head space (660 ml). Using Henry’s constants at 37 °C [31], 1 mbar corresponds to 2.6 × 10^−6^ mol CO_2_, 6.6 × 10^−6^ mol H_2_S or 1.1 × 10^−7^ mol O_2_ in the solution (100 ml). Some small amount of dissolved CO_2_ (less than 1%) will react to form carbonic acid H_2_CO_3_. With a pK_a1_ = 6.37, about 90% of this carbonic acid will dissociate to the bicarbonate ion HCO_3_^−^ at the pH range of the bacterial suspension (6.9–7.2). Since only about 10% of the total CO_2_ is in solution, of which only a small amount will be converted into carbonic acid, this loss of CO_2_ can be neglected to a first approximation. Being an acid, dissolved H_2_S can dissociate into HS^−^; with a pK_a1_ = 7.02, about half of dissolved H_2_S will dissociate at the pH range of the suspension. With about 25% of total H_2_S being in solution, of which about 50% is lost in the form of HS^−^, this effect needs to be considered for the total sulfur balance arising from the conversion of cysteine.

**Fig. 7:**
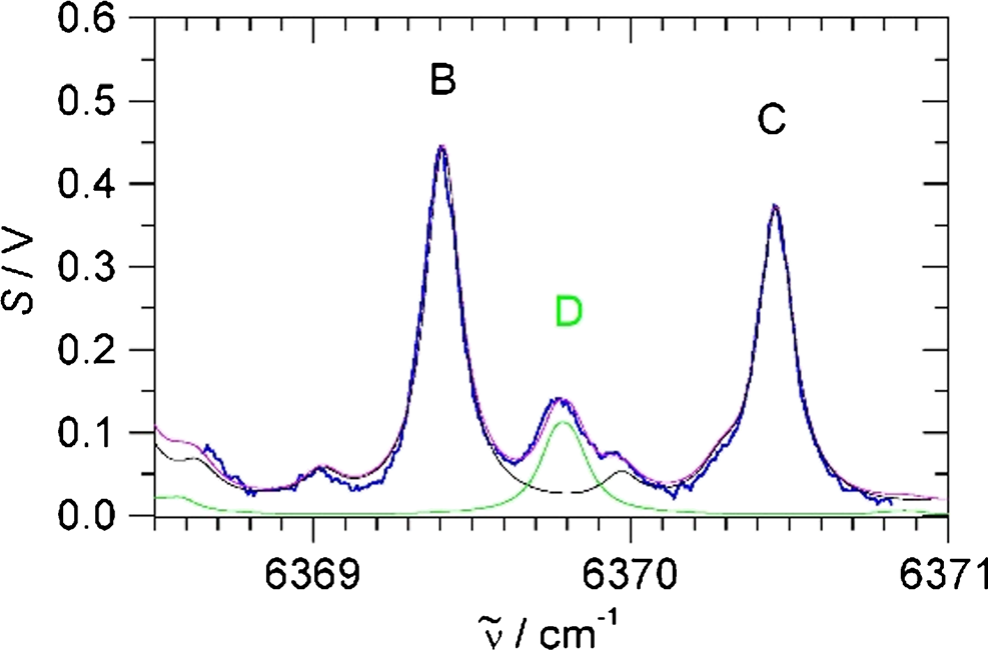
Typical scan of the head space above a bacterial suspension (2 h after injecting 1 mM cysteine, see Fig. 8). The blue trace is the photoacoustic signal. In black, green and magenta are HITRAN data for 98 mbar CO_2_, 2.2 mbar H_2_S and the sum, respectively. The peak labels are as in Fig. 4

In the experiment, we monitored O_2_ with the red diode and converted the photoacoustic signal into millibar using the previous calibration. For simultaneous monitoring of CO_2_ and H_2_S, we used the near-IR diode scanning between 6369 and 6371 cm^−1^. In this range, the CO_2_ peaks labelled **B** and **C** can be found, and in-between, the strong H_2_S peak labelled **D**. Figure 7 shows a typical scan of the head space above the bacterial suspension. Fitting three Lorentzian curves to the observed peaks **B**, **C** and **D** and using the previous calibration, photoacoustic signal is converted into millibar of H_2_S and CO_2_. Measurements were repeated every 15 min to obtain time-dependent concentrations of the head space.

**Fig. 8:**
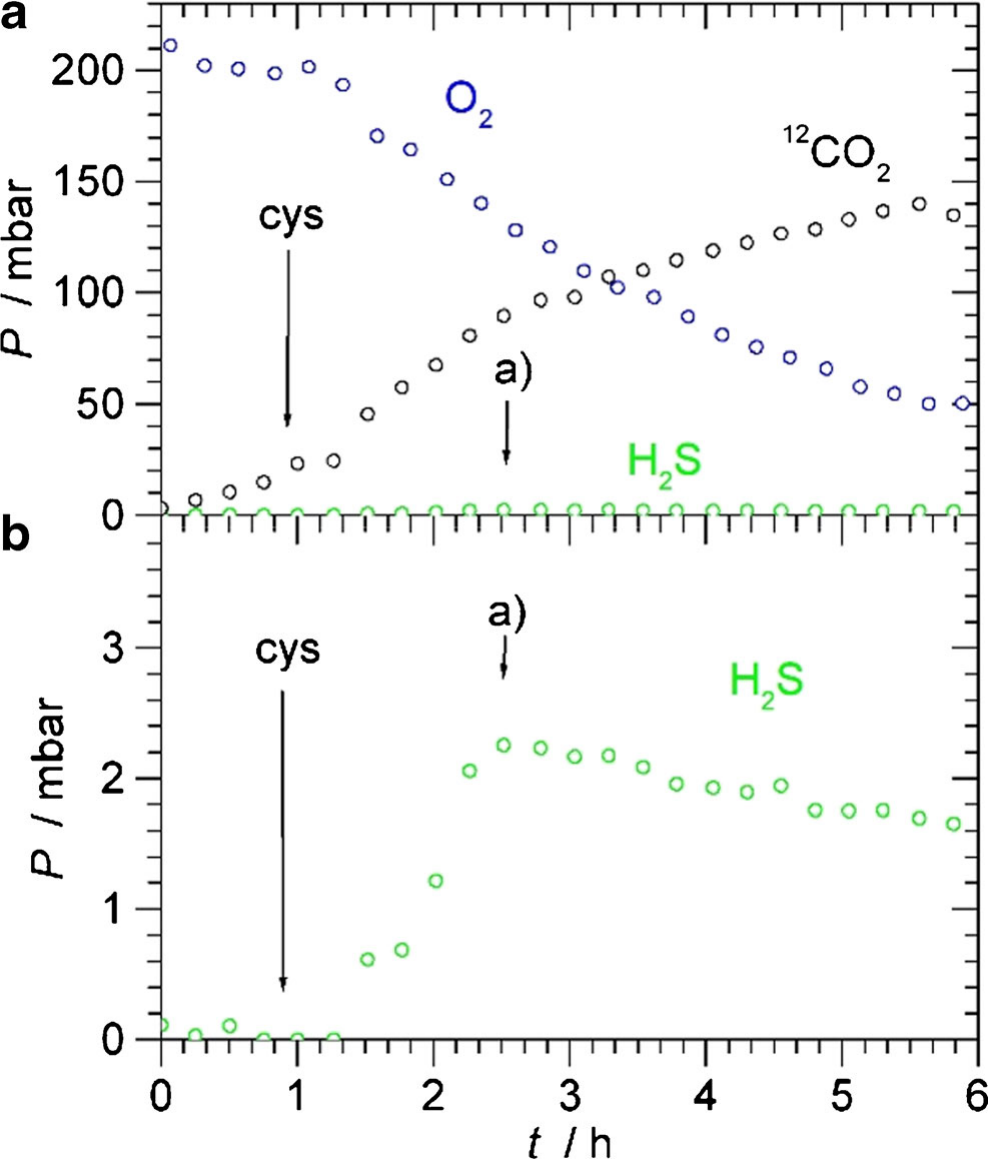
**a**, **b** Time-dependent concentrations of O_2_, CO_2_ and H_2_S in the head space during the metabolism of 1 mM l-cysteine by *E*. *coli* in 100 ml 2YT. **b** has an expanded pressure scale to show the H_2_S partial pressure more clearly. “cys” denotes the time of injection of the cysteine

Figure 8 shows a typical example of time-dependent concentrations, where 1 mM cysteine was injected into the *E*. *coli* suspension in 2YT medium after a delay of about 1 h. Before injection, no traces of H_2_S are apparent. Due to aerobic respiration of *E*. *coli*, O_2_ is slowly decaying and CO_2_ formed in an about 1:1 ratio, as expected. After injection of cysteine (labelled “cys” in Fig. 8), metabolism accelerates, and H_2_S is formed after a short lag phase of about 20 min. Within 1 h, H_2_S peaks to about 2.2 mbar. Apparently, all cysteine is converted by then, and H_2_S concentrations fall very slowly afterwards. The 2.2 mbar H_2_S corresponds to about 0.09 mmol in the head space and suspension. This is almost exactly the amount of cysteine injected, demonstrating complete conversion of cysteine within 1 h. The slow decay of H_2_S is genuine and not due to leaks, since we do not observe decays for CO_2_. H_2_S is known to stick to surfaces and is easily adsorbed which may explain the decaying pressure. Experiments were repeated several times, including utilising different concentrations of cysteine (1–4 mM). In all cases, the same qualitative behaviour was observed, with complete conversion of cysteine into H_2_S occurring within about 1 h. In a control experiment, we injected cysteine into sterile 2YT medium without observing any H_2_S evolving, proving that the H_2_S production is due to microbial activity. The current application demonstrates in situ monitoring of trace gases in the head space above bacterial suspensions in a closed system by Helmholtz photoacoustic detection. In future work, this method can be employed to study H_2_S production by microbes and how it is affected and modulated by different media, feed stock and gene expressions, and its role as signalling molecule and in the defence against antibiotics.

### Detection of H_2_S and CO_2_ impurities in natural gas samples

Trace gas analysis of natural gas is a very relevant task in petrochemistry. Natural gas is mainly methane, but depending on the source or provenance, it may also contain minor components like higher alkanes, components of no caloric value such as N_2_ and CO_2_ and even toxic compounds like H_2_S. “Sour” gas streams contain particularly high levels of CO_2_ and H_2_S which present a danger to equipment and pipelines and to human health. Due to its high toxicity, H_2_S detection is of special concern and it must be removed from the gas stream before the gas can be utilised. To demonstrate the capability of our scheme for trace gas detection of H_2_S and CO_2_ impurities in natural gas samples, we have sampled natural gas from a gas tap within the Department and scanned the near-IR photoacoustic spectrum between 6369 and 6372 cm^−1^. In this region, there are CO_2_ transitions (peaks labelled **B** and **C** in Figs. 4 and 7) and a separated H_2_S transition (labelled **D**). In 1 bar of natural gas, the acoustic resonance frequency shifts to about 320 Hz, and the molecules have also slightly different phase shifts compared to 1 bar air or N_2_. We have verified that after optimization, essentially the same calibration, noise levels and detection limits as shown above for N_2_ apply, in particular a 25 ppmv noise equivalent detection limit for H_2_S in 1 bar natural gas at 1-s integration time.

**Fig. 9:**
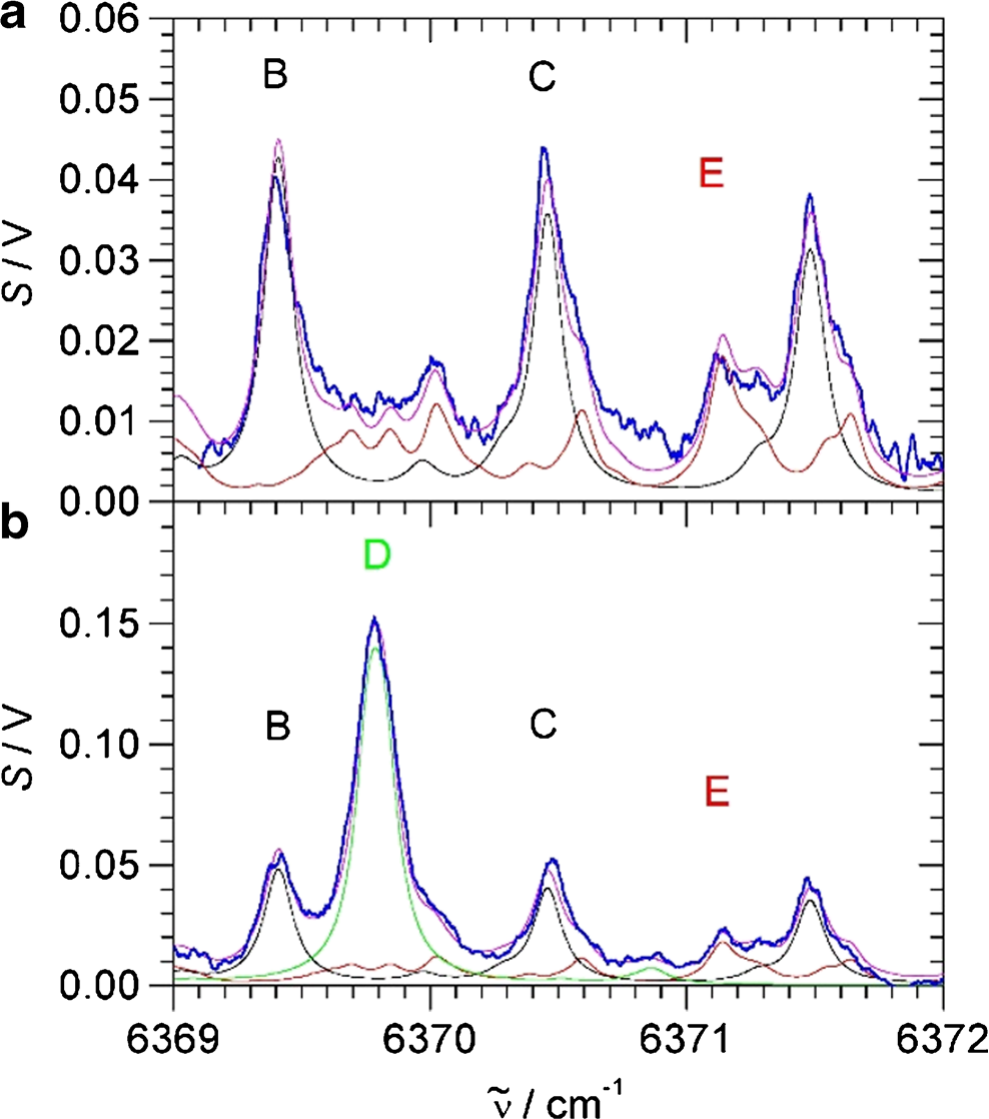
CH_4_, CO_2_ and H_2_S transitions of natural gas samples. **a** 1 bar natural gas (sampled 29 October 2018). **b** 3 mbar H_2_S in 1 bar natural gas (sampled 7 November 2018). The blue trace is the photoacoustic signal. In red, black, green and magenta are HITRAN data for CH_4_, CO_2_, H_2_S and the sum, respectively. The peak labels are as in Fig. 4 (see main text for more details)

Figure 9a) shows a scan of 1 bar natural gas; in the spectral region, there are weak CO_2_ transitions due to natural CO_2_ impurities (**B** and **C** in Fig. 9a). In addition to a very weak H_2_O absorption peak at the position of the H_2_S peak **D** discussed above, there are also weak CH_4_ absorptions with *σ*_peak_ = 1.5 × 10^−5^ pm^2^ [30], about 5000 times weaker than H_2_S. Assuming a similar sensitivity of photoacoustic signals to methane, the noise limit will be exceeded at methane pressures above 100 mbar. Unlike water discussed above, this must be considered in natural gas samples, where at 1 bar total pressure, methane will typically be between 900 and 1000 mbar. In the spectrum of Fig. 9a), these CH_4_ transitions at the position of the H_2_S have a peak value of about 10 mV, above ten times the noise level at 1-s integration time. To correct for these weak absorptions, we suggest extending the spectral region to include the separate peak **E** of methane to establish the content of CH_4_, and then subtract methane proportionally at the position of the H_2_S, as demonstrated in Fig. 9. A comparison with the HITRAN database and our previous calibration shows the presence of 14 mbar CO_2_ in the 1 bar sampled natural gas, or 1.4% CO_2_ content, very similar to a previously reported measurement using Raman detection [4]. Reassuringly, no H_2_S is apparent within our detection limit. To demonstrate the sensitivity to H_2_S detection, we have prepared a sample of 3 mbar H_2_S in 1 bar natural gas. The measurement is shown in Fig. 9b). The methane background is essentially as in pure natural gas. According to the calibration, there is 16 mbar or 1.6% CO_2_, very close to the previous measurement of pure natural gas; the slight deviation might be explained by purity fluctuations in the natural gas line. The measured H_2_S content is 2.8 mbar, very close to the nominally 3 mbar as prepared. In future work, we would like to sample natural gas at different sources including natural H_2_S impurities, to demonstrate the full potential of our detection scheme for monitoring toxic gases in the petrochemistry.

## Conclusions

Due to the inherent signal amplification and noise cancellation, photoacoustic spectroscopy in a differential Helmholtz resonator has a great potential for trace gas analysis, with possible applications including safety monitoring of toxic gases and applications in the biosciences and for natural gas analysis in petrochemistry. In this study, we describe a setup employing near-IR and red diode lasers for the detection of CO_2_, H_2_S and O_2_ in 1 bar of air/N_2_ and natural gas, in static and flow cell measurements and introduce improvements including a multipass arrangement and using two independent lasers. With the red diode laser, O_2_ can be detected at 764.280 nm (vacuum). A noise equivalent detection limit of 0.60 mbar (600 ppmv) of O_2_ in 1 bar of air at 35-mW peak power and 1-s integration time is obtained. Normalising to the absorption cross section, the laser power and integration time, a noise equivalent normalised absorption coefficient *α* = 2.2 × 10^−8^ cm^−1^ W s^1/2^ is calculated. Within the tuning range of the near-IR DFB diode laser (6357–6378 cm^−1^), CO_2_ and H_2_S absorption features can be accessed for trace gas detection, with a noise equivalent detection limit of 0.160 mbar (160 ppmv) CO_2_ in 1 bar N_2_ at 1-s integration time with the 30-mW laser. This corresponds to a noise equivalent normalised absorption coefficient *α* = 8.3 × 10^−9^ cm^−1^ W s^1/2^. Due to stronger absorption cross sections, the noise equivalent detection limit of H_2_S in 1 bar N_2_ is 0.022 mbar (22 ppmv). At this level, the scheme may be useful for safety monitoring of toxic H_2_S. Similar detection limits apply to trace impurities in 1 bar natural gas. Detection limits scale linearly with laser power and with the square root of integration time. At 16-s total measurement time to obtain a spectrum, a noise equivalent detection limit of 40 ppmv CO_2_ is obtained after a spectral line fitting procedure, for example. Considering the simplicity of the DHR setup, the detection limits compare favourably with more involved photoacoustic schemes. Possible interferences due to weak water and methane absorptions have been discussed and shown to be either negligible or easy to correct. We have demonstrated two selected application examples. The setup has been used successfully for simultaneous in situ monitoring of O_2_, CO_2_ and H_2_S in the biosciences, for example in the cysteine metabolism of microbes (*E*. *coli*), and for the analysis of CO_2_ and H_2_S impurities in natural gas. Using Henry’s law, the 1-s noise equivalent detection limits for the head space gases O_2_, CO_2_ and H_2_S translate into detection limits of the dissolved gases in the solution of 16 ppb per mole for dissolved O_2_, 76 ppb for CO_2_ and 27 ppb for H_2_S.

In the future, we want to explore the possibility of isotope labelling experiments taking advantage of the different spectroscopic signatures of isotopes. We are planning further work to miniaturise the setup and to apply the technique to the measurement of natural gas samples from different sources and to investigate the metabolism of microbes with applications in studying the role of H_2_S as a signalling molecule in microbial systems. Due to the low cost of diode lasers and microphone detection, the relatively simple and robust setup, the inherent signal amplification and noise cancellation, the suitability for static and flow cell measurements, the good detection limits and the spectroscopic selectivity which minimises interferences, trace gas analysis in a differential Helmholtz resonator with near-IR and red diode lasers has a great potential for many applications ranging from the biosciences to safety monitoring to petrochemistry.
